# MicroRNA-206 is differentially expressed in *Brca1*-deficient mice and regulates epithelial and stromal cell compartments of the mouse mammary gland

**DOI:** 10.1038/oncsis.2016.27

**Published:** 2016-04-04

**Authors:** A Wronski, G K Sandhu, M J G Milevskiy, B L Brewster, J A Bridge, A M Shewan, S L Edwards, J D French, M A Brown

**Affiliations:** 1 School of Chemistry and Molecular Biosciences, University of Queensland, St Lucia, Queensland, Australia; 2 QIMR Berghofer Medical Research Institute, Brisbane, Queensland, Australia

## Abstract

Depletion of Brca1 leads to defects in mouse mammary gland development and mammary tumors in humans and mice. To explore the role of microRNAs (miRNAs) in this process, we examined the mammary glands of *MMTV-Cre Brca1*^*Co/Co*^ mice for differential miRNA expression using a candidate approach. Several miRNAs were differentially expressed in mammary tissue at day 1 of lactation and in mammary epithelial cell lines in which *Brca1* messenger RNA (mRNA) levels have been reduced. Functional studies revealed that several of these miRNAs regulate mammary epithelial cell function *in vitro*, including miR-206. Creation and analysis of MMTV-miR-206 transgenic mice showed no effect on lactational mammary development and no tumors, but indicates a role in mammary tissue remodeling in mature mice, potentially involving *Igf-1* and *Sfrp1*. These results indicate the potential of miRNAs to mediate the consequences of *Brca1* loss and suggest a novel function for miR-206.

## Introduction

Mutations in the breast cancer susceptibility gene *BRCA1* significantly increase the risk of developing breast cancer. Although the many characteristics of BRCA1-associated tumors have been elucidated, the early molecular changes that arise as a consequence of disruption of *BRCA1* expression in the mammary gland and lead to mammary tumorigenesis are not well understood.

Targeted repression and/or deletion of *Brca1* transcript leads to aberrant lobular–alveolar development *in vivo*,^1, 2^ and defective mammary epithelial differentiation *in vitro*.^3, 4^ Notably, tumors arising from conditional *Brca1*-knockout mice are reminiscent of human *BRCA1*-associated breast cancers sharing similar morphological and molecular characteristics, and clustering with the basal molecular subtype.^5, 6, 7, 8, 9, 10^ The molecular basis of these phenotypes however, is yet to be defined. To elucidate the molecular consequences of *Brca1* depletion in the mouse mammary gland, we have previously investigated the role of coding genes in *Brca1* loss. This analysis identified many genes differentially expressed in conditional *Brca1*-knockout mice at day 1 of lactation, including the receptor tyrosine kinase, *c-Kit*, which marks luminal progenitor cells in preneoplastic *BRCA1* mutation carrier tissue and may mediate the expansion of luminal progenitor cells in *BRCA1* loss.^11, 12, 13^ This supports the hypothesis that luminal cells are the cells of origin of *BRCA1*-associated tumors^14, 15, 16, 17^ and that *BRCA1* may have a role in regulating mammary epithelial cell fate (reviewed in reference 18).

We now investigate the role of miRNAs in *Brca1*-deficient mouse mammary glands. miRNAs are small evolutionary-conserved RNAs that are implicated in many biological processes and diseases (reviewed in reference 19). miRNAs are differentially expressed during mammary gland development^20, 21^ and regulate the expression of milk transcripts,^22, 23^ self-renewal of mammary epithelial progenitor cells,^24^ ductal outgrowth^25^ and the modulation of key transcriptional networks underpinning the development of the mammary gland.^25, 26, 27^ The role of these molecules in *Brca1*-associated mammary epithelial defects however is unknown. In this study, we demonstrate the effects of *Brca1* deficiency on the expression of miRNAs in the mouse mammary gland using both *in vitro* and *in vivo* models. We show that *Brca1* loss in the lactating mammary gland results in the differential expression of miRNAs, and explore their roles in mammary gland morphogenesis.

## Results and discussion

### Identification of miRNAs that are differentially regulated in the mammary glands of *MMTV-Cre Brca1^Co/Co^* mice

To determine the impact of *Brca1* deficiency on miRNA expression in the mouse mammary gland, we assessed the levels of nine miRNAs from *MMTV-Cre Brca1^Co/Co^* mice mammary glands at day 1 of lactation. These miRNAs were selected using a candidate approach with the following selection criteria: (i) expressed in breast tumors, in particular, tumors associated with *BRCA1* mutation/repression (triple negative or basal subtype)^28^ and (ii) differentially expressed during mouse mammary gland development, in particular, those that inversely mirror the expression of *Brca1*.^21^

**Figure 1:**
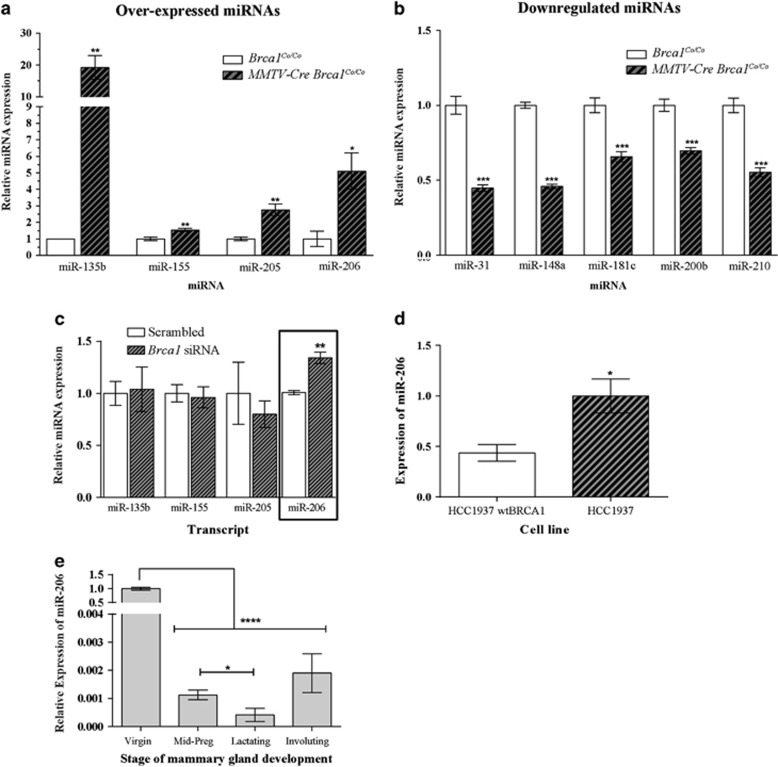
miR-206 is differentially expressed in conditional *Brca1*-knockout mice mammary glands and during mammary gland development. (**a**, **b**) miRNAs are differentially expressed in conditional *Brca1*-knockout glands at day 1 of lactation. Total RNA from *Brca1^Co/Co^* and *MMTV-Cre Brca1^Co/Co^* mouse mammary glands (*n*=8 per genotype) as previously described^19^ was used for quantitative real-time PCR (qRT-PCR) analysis. Complementary DNA was generated using the Qiagen miScript Reverse Transcription kit (Qiagen GmbH, Hilden, Germany) using 10–200 ng of RNA as input. Reactions were run on a Qiagen Q Rotorgene machine with the Qiagen miScript SYBR Green kit as per manufacturer's instructions. Qiagen miScript Primer Assays were used for miR-31 (MS00001407), miR-135b (MS00001575), miR-148a (MS00001652), miR-155 (MS00001701), miR-181c (MS00011277), miR-200b (MS00011417), miR-205 (MS00003780), miR-206 (MS00001869), miR-210 (MS00001890) and RNU6b (MS00014000). RNU6b was used as an internal housekeeping control and no template and water controls were run concurrently to ensure no contamination present. Differential gene expression was determined using the ΔΔCt method. Each bar represents mean expression±s.e.m., compared with control. (**c**) miR-206 is overexpressed in mouse mammary epithelial cells that have reduced *Brca1* expression. HC11 cells, a mouse mammary epithelial cell line, a kind gift from Chris Ormandy (Garvan Institute, Sydney, NSW, Australia) were cultured in RPMI-1640 media (Thermo Fisher Scientific, Waltham, MA, USA), supplemented with 10% fetal bovine serum (Thermo Fisher Scientific), 5 μg/ml insulin and 10 ng/ml recombinant epidermal growth factor (rEGF), both from Sigma Aldrich (St Louis, MO, USA) and antibiotic–antimycotic (Thermo Fisher Scientific). HC11 cells were transfected with Brca1 synthetic RNA (siRNA) using Lipofectamine 2000 (Thermo Fisher Scientific) as per manufacturer's instructions. SMARTpools of siRNA (Thermo Fisher Scientific) targeting mouse Brca1 (L-040545-00) and a nontargeting control (D-001810-10) were used at a concentration of 100 nm to transfect HC11 cells at subconfluence. Total RNA was extracted using TRIzol Reagent (Thermo Fisher Scientific) and analyzed for miRNA expression as described above. (**d**) miR-206 is overexpressed in human mammary epithelial cells that harbor a BRCA1 mutation. HCC1937 cells and their wtBRCA1 control were assayed for miR-206 expression using qRT-PCR as described above. HCC1937 cells were cultured in RPMI media supplemented with 10% fetal bovine serum, 1 mm sodium pyruvate and antibiotic–antimycotic (Thermo Fisher Scientific). (**e**) miR-206 is differentially expressed during mouse mammary gland development. Total RNA from virgin (5-week-old), day 15 pregnancy (midpregnant), day 1 of lactation (lactating) and day 5 of involution (involuting) was extracted using a Qiagen miRNeasy Mini Kit (Qiagen), and were used to determine miR-206 expression as described above. Statistical significance was determined by Student's *t*-test and denoted as ****P<0.0001, ***P<0.001, **P<0.01 and *P<0.05. Throughout the manuscript, the number of samples for each experiment was determined using experimental guidelines and at least three biological replicates. For mice experiments, a power analysis was used. No samples were excluded from analysis and variation was similar among samples. All cell lines are regularly tested for mycoplasma.

Out of the nine miRNAs that were screened, four were upregulated (miR-135b, miR-155, miR-205 and miR-206: Figure 1a) and five were downregulated (miR-31, miR-148a, miR-181c, miR-200b and miR-210: Figure 1b). Upregulation of miR-155, a known oncomiR,^29^ is interesting, given that it has previously been reported to be overexpressed in *BRCA1*-associated breast cancers and to be transcriptionally repressed by BRCA1.^30^ Three of the downregulated miRNAs (miR-148a, miR-181c and miR-210) are normally highly expressed during lactation in the mouse mammary gland.^21^ As *Brca1* depletion in these mice results in a defect in lobular–alveolar differentiation,^1^ we speculate that reduced expression of these miRNAs could contribute to the phenotype observed in *Brca1*-deficient mammary glands.

To explore the potential functional consequences of the observed differential expression of miRNAs, we conducted *in silico* analysis of their predicted mRNA targets. We used the m3RNA algorithm^31^ to identify putative miRNA target genes (Supplementary Table 1) and utilized Ingenuity Pathway Analysis to infer functional consequences (Supplementary Table 2). We compared a previously generated list of coding genes that were differentially expressed in the mammary glands of conditional *Brca1*-knockout animals vs controls at day 1 of lactation,^11^ with the predicted mRNA targets of the miRNAs altered in the same tissues (Figures 1a and b). This generated a list of mRNAs whose expression is inversely correlated to the expression of the miRNA and are thus potential targets of these differentially expressed miRNAs. Interestingly, in both instances where miRNAs are downregulated and mRNAs upregulated and vice versa, cancer is a key disease category that is influenced, suggesting that these miRNA–mRNA interactions may contribute to tumorigenesis (Supplementary Table 2).

To investigate if the overexpression of miRNAs observed in lactating mammary tissue of *MMTV-Cre Brca1*^*Co/Co*^ were also evident in differentiation-competent mammary epithelial cells with reduced levels of *Brca1,* we assessed miRNA expression in HC11 cells in which *Brca1* expression was reduced. This demonstrated that out of the four miRNAs assessed, only miR-206 expression was significantly altered (Figure 1c). Consistent with this, we also found that the expression of miR-206 was increased in HCC1937 cells, which contain a *BRCA1* germline mutation resulting in reduced *BRCA1* expression and a truncated BRCA1 protein^32^ as compared with the wt*BRCA1* control (Figure 1d).

In contrast to the analysis of *Brca1*-deficient mammary glands (Figure 1a), upregulation of miR-135b, miR-155 and miR-205 was not observed in HC11 cells in which *Brca1* levels have been repressed using siRNA (Figure 1c). There are several possible explanations for this including the higher levels of *Brca1* repression using gene deletion (*in vivo*) compared with siRNA (*in vitro*) and the heterogeneous cellular environment in mouse mammary tissue compared with a relatively homogenous cell line. For example, it is possible that repression of *Brca1* in the epithelial compartment of the mammary gland causes upregulation of miR-135b, miR-155 and miR-205 in nonepithelial cells of the mammary gland. There are many examples of disease-associated miRNAs with altered expression in the stromal compartment of the mammary gland^33, 34^ and miR-155 has previously been implicated in the transformation of stromal fibroblasts.^35^

The expression of miR-206 was also evaluated in mouse mammary epithelial tissue at various stages of the mammary gland development (virgin, pregnant, lactating and involution). miR-206 levels were highest in virgin animals as compared with the other stages of mammary gland development (Figure 1e). Interestingly, this is the opposite of what is observed with *Brca1* during the mammary gland development.^36^ Given the role of miR-206 in breast cancer and that it is upregulated in *Brca1* loss, it is possible that it may contribute to the consequences of *Brca1* loss in the mouse mammary gland.

**Figure 2:**
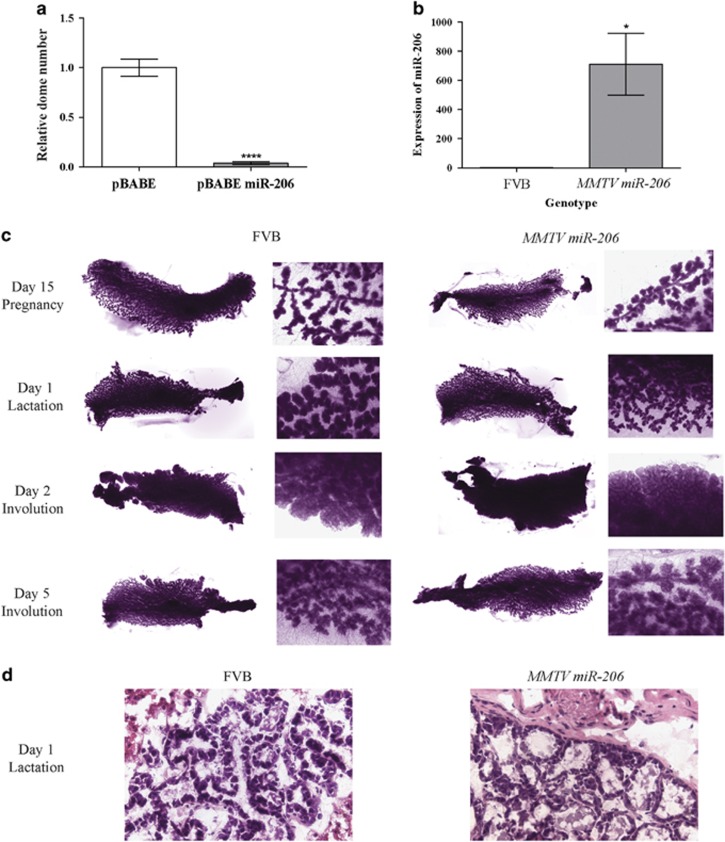
Overexpression of miR-206 diminishes the differentiative capacity of HC11 cells, but does not affect mammary gland development. (**a**) Overexpression of miR-206 reduces the ability of HC11 cells to form domes *in vitro*. HC11 cells were transduced with pBABE or pBABE miR-206 virus, created by transfecting BOSC23 cells. Stable expressing HC11 cell lines were selected for using puromycin (Clontech Laboratories, Mountain View, CA, USA). Cells were then plated into the HC11 dome assay. Subconfluent cells in a six-well plate were cultured in recombinant epidermal growth factor (rEGF)-free HC11 media for 48 h. The media was then changed to include 100 nm dexamethasone and 5 μg/ml ovine prolactin (both from Sigma Aldrich, St Louis, MO, USA) in rEGF-free HC11 media for a further 72 days. During this time, the media was replaced every 48 h. The number of domes was then counted manually, as observed using a light microscope on day 8 of the assay. Each bar represents mean expression±s.e.m., compared with control of three independent experiments. (**b**) miR-206 is overexpressed in MMTV miR-206 glands. To create MMTV miR-206 mice, a linearized pMMTV miR-206 plasmid was used for pronuclear injection of FVB/NJ embryos (JAX stock No. 001800) by the Transgenic Animal Service (TASQ – University of Queensland, Brisbane, QLD, Australia). All subsequent pups were genotyped by the Australian Equine Genetics Research Centre (AEGRC; University of Queensland, Brisbane, QLD, Australia). Females were time-mated and killed at either day 5 of pregnancy, day 1 of lactation, day 2 of involution or day 5 of involution for further analysis. Expression of miR-206 in MMTV miR-206 animals was evaluated using qRT-PCR as described earlier. At least three animals were assayed in triplicate and expression normalized to FVB control animals. All animal experiments were approved by the University of Queensland Animal Ethics Committee; AEC Approval Number: SCMB/273/11/CCQ. (**c**) MMTV miR-206 animals do not show any anatomical differences during mammary gland development. The right abdominal glands were carefully spread onto a glass slide and fixed in Carnoy's fixative (six parts 100% ethanol, three parts chloroform and one part glacial acetic acid) for wholemount analysis. These were then stained in carmine alum stain until the stain penetrated the entire gland. Wholemounts were then visualized with a Nikon stereomicroscope and imaged with a Nikon DS-Fi1c digital microscope camera (both from Nikon Instruments, Melville, NY, USA). (**d**) No changes observed during day 1 of lactation in MMTV miR-206 glands. Left thoracic glands were flash frozen in Tissue-Tek Optimal Cutting Temperature (OCT) compound (Sakura, Netherlands) and sections were cryosectioned to 10 μm thickness and stained with hematoxylin and eosin by the School of Biomedical Sciences Histology Laboratory (University of Queensland, Brisbane, QLD, Australia). Sections were visualized on a Zeiss Axiophot 2 (Carl Zeiss, Oberkochen, Germany) and imaged with a SPOT color camera (SPOT Imaging Solutions, Sterling Heights, MI, USA). All imaging results are representative images of at least three animals per genotype. Mice were not randomized as they were required to be a certain genotype. The experimenter was blinded to the genotype when assessing phenotype throughout.

Loss of *Brca1* transcript expression leads to decreased proliferation,^37^ increased apoptosis^1, 38^ and defects in the ability to undergo both *in vitro* and *in vivo* mammary epithelial differentiation.^1, 2, 3, 4, 11^ Interestingly, miR-206 overexpression results in defective mammary epithelial differentiation (Figure 2a), *in vitro* proliferation defects^39, 40, 41^ and the induction of apoptosis via the repression of *Notch3*.^42^ These results together with the differential expression data above suggest miR-206 is a strong candidate acting downstream of *Brca1*. We therefore decided to further explore the role of miR-206 in mammary gland development.

### Overexpression of Brca1-associated miRNAs affects mammary epithelial morphogenesis *in vitro*

To address the hypothesis that miRNAs overexpressed in *Brca1*-deficient mammary tissue may affect mammary epithelial morphogenesis, we determined the effect of ectopic overexpression for each miRNA in HC11 cells. Overexpression of miR-155, miR-205 and miR-206 resulted in a complete loss of HC11 dome formation, whereas, overexpression of miR-135b resulted in an increase in HC11 dome formation (Supplementary Figures 1a and b). A role for miR-155, miR-205 and miR-206 in mammary epithelial morphogenesis is consistent with previous studies in other epithelial cell types.^43, 44, 45^

### MMTV-miR-206 mice show a defect in mammary gland structure after 12 months

Given that miR-206 is upregulated in the mammary glands of *Brca1* conditional knockout mice and in HC11 cells treated with a *Brca1* siRNA, and limits the ability of HC11 cell morphogenesis *in vitro*, we prioritized miR-206 for *in vivo* analysis to further explore its role in mammary gland development and function. Transgenic mice expressing miR-206 under the control of the MMTV promoter were created and expression was confirmed in mammary glands at day 1 of lactation (Figure 2b). Analysis of the glands revealed no apparent differences in gross morphology or tissue architecture during pregnancy, lactation or involution (Figures 2c and d). This suggests either that miR-206 has no role in these processes or that any affect is masked by increased branching morphogenesis induced by the various hormonal cascades during pregnancy and lactation.^46^

**Figure 3:**
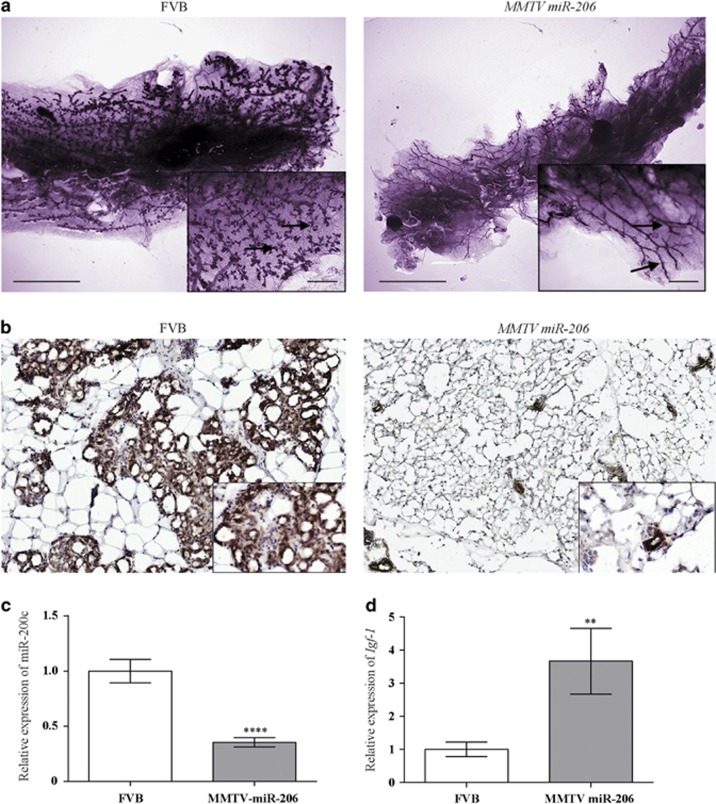
Aged MMTV miR-206 mammary glands exhibit a reduction in epithelial density and an increase in markers of accelerated aging. (**a**) Aged MMTV miR-206 animals have reduced branching structures. FVB and MMTV miR-206 animals were aged to 12–15 months and killed. Wholemount analysis was carried out as described in Figure 2. Scale bars indicate 0.67 and 1 μm in inset image. (**b**) Aged MMTV miR-206 animals have reduced beta-catenin expression. Left thoracic glands were snap frozen as above and cryosectioned. Immunohistochemical staining of beta-catenin was carried out by QIMR-Berghofer histology services using an anti-rabbit beta-catenin antibody (Eptiomics Inc., Clone #E247) at 1:500. All imaging results are representative images of at least three animals per genotype. (**c**) Aged miR-206 mammary glands display a reduction in the epithelial marker, miR-200c. Total RNA from the mammary glands of aged FVB controls or MMTV miR-206 animals was isolated and expression of miR-200c analyzed by qRT-PCR as described in Figure 1. (**d**) Aged miR-206 mammary glands have increased secreted stromal factor, *Igf-1* expression. Expression of *Igf-1* was assessed using TaqMan Universal PCR Master Mix (Thermo Fisher Scientific), with complementary DNA generated using SuperScript III (Thermo Fisher Scientific) as per manufacturer's instructions and TaqMan probes for *Igf-1* (Mm00439560_m1). *Hprt* was used as a housekeeping control (TaqMan Probe: Mm01545399_m1). Statistical significance was determined by Student's *t*-test and denoted as ****P<0.0001 and **P<0.01.

In contrast, MMTV miR-206 transgenic mice at 12–15 months of age displayed a striking mammary phenotype. This was characterized by a significant increase in fatty tissue and a significant reduction of branching within the mammary epithelial tree, suggestive of tissue degeneration, in all of the MMTV miR-206 mice (*n*=5) and one of the six control mice (Figure 3a). No evidence of mammary tumors was observed prior to the end of study. Interestingly, several transgenic mouse models of *Brca1* display a similar phenotype.^47, 48, 49, 50, 51^ Jones *et al.*^50^ argue that the *Brca1* phenotype is due to the action of increased estrogen/IGF-1 on the mammary stroma microenvironment and that this may create an environment that is permissive to tumor development. The late onset of tumor development in mouse models of *BRCA1* disruption is consistent with this.^9^ In support of this hypothesis, *BRCA1* negatively regulates IGF-1 and loss of *BRCA1* is associated with an increase in IGF-1.^52^ A plausible hypothesis is therefore that loss of *Brca1* causes an increase in miR-206, which in turn results in tissue remodeling in older mice and that this permits *Brca1*-associated tumor development.

There are several potential molecular and cellular mechanisms for the observed phenotype, including a miR-206-associated increase in epithelial cell apoptosis, epithelial to mesenchymal transition, induction of IGF and/or adipose production. To explore this, we first used qRT-PCR to confirm that miR-206 was still overexpressed in the mammary glands of transgenic mice at 12 months of age. Interestingly, higher miR-206 expression corresponded to a stronger phenotype, concurrent with this, we found elevated levels of miR-206 in the single control mouse that displayed the phenotype (data not shown). We then examined the expression of epithelial and mesenchymal compartment markers within the aged mice mammary glands. β-catenin staining that detects the epithelial content of the mammary gland, revealed lesser ductal and end bud structures in the MMTV miR-206 glands as compared with the controls (Figure 3b). The expression of miR-200c, another epithelial marker, was found to be significantly decreased in MMTV miR-206 glands as compared with the controls, further corroborating the β-catenin staining (Figure 3c).

To explore the possibility that the IGF-1 pathway may also contribute to the phenotype in miR-206 mice, the expression of *Igf1* was evaluated and normalized against the total volume of epithelial cells using miR-200c.^53, 54, 55^ The results revealed that *Igf1* was significantly upregulated in the MMTV miR-206 glands as compared with the controls (Figure 3d). This supports the hypothesis that the *Igf1* pathway may contribute to the phenotype in MMTV-miR-206 mouse mammary glands. As *Igf1* is a secreted stromal factor that signals in a paracrine manner to the epithelial cell, this also raises the possibility that miR-206 induces an expansion of the stromal compartment, which could explain the apparent higher fat content seen in the MMTV miR-206 mammary glands as compared with the controls.^56, 57, 58^ Interestingly, mature *Brca1*-deficient mice also demonstrate an increase in mammary adipose tissue.^50^

Although the proposed expansion of the stromal compartment may promote tumorigenesis, no tumors were detected in the mammary glands of MMTV-miR-206 glands. It is possible that this reflects a dependence on other components of the *Brca1* pathway or on intact p53 activity, which when lost rescues cellular aging but induces tumorigenesis.^55^

### miR-206 targets the 3′UTR of the Wingless-type MMTV integration site (Wnt) antagonist, Sfrp1

**Figure 4:**
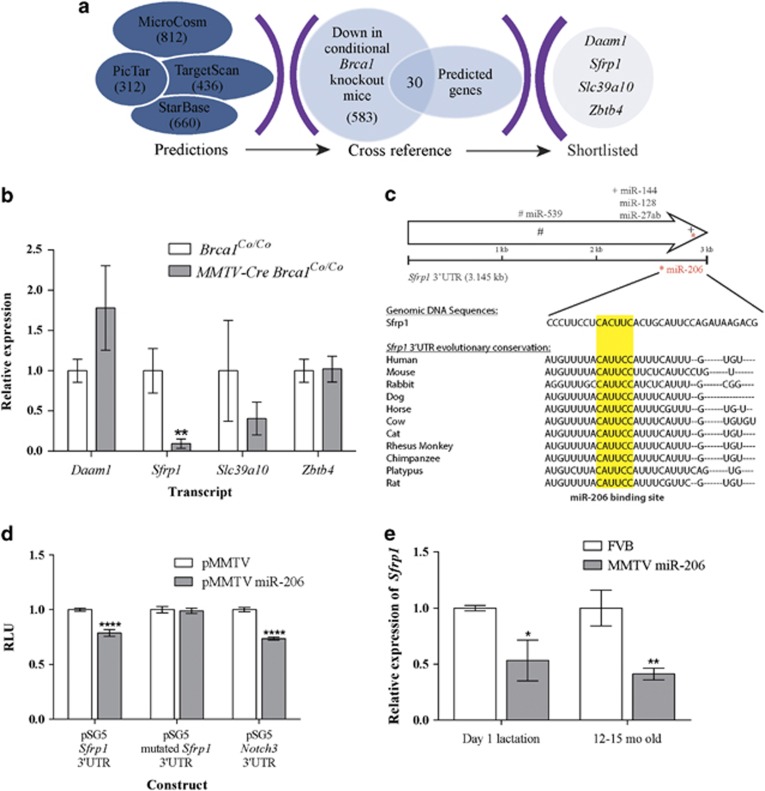
miR-206 targets the Wnt antagonist, *Sfrp1*. (**a**) Schematic of miRNA target gene prediction. miRNA targets were predicted using MicroCosm (http://www.ebi.ac.uk/enright-srv/microcosm/htdocs/targets/v5/), PicTar (http://pictar.mdc-berlin.de/), TargetScanMouse (http://www.targetscan.org/mmu_61/) and StarBase (http://starbase.sysu.edu.cn/). Genes were then cross-referenced with the downregulated genes identified in reference 19 and four genes were shortlisted. (**b**) A predicted miR-206 target, *Sfrp1* is downregulated in lactating conditional *Brca1*-knockout animals. RNA from *Brca1*^*Co/Co*^and *MMTV-Cre Brca1*^*Co/Co*^ animals was collected as described earlier. qRT-PCR was used as described above to assess the expression of *Daam1* (For: CATTGATCAGCTCAATTCCATGG, Rev: TGCTGTCTTCAGACTCTCGATGG), *Sfrp1* (For: AATACCACGGAAGCCTCTAAGCC, Rev: TTGCACAGAGATGTTCAATGATGG), *Slc39a10* (For: AGTAGGAACAATGAGTGGAGACGC, Rev: GAATGACCATGTCCGTGCG) and *Zbtb4* (For: TGAGAAGGTGTTTGCCCTGG, Rev: TAGGTGACAAAGGTGTCCCAGC). *Hprt* was used as a housekeeping gene (For: GCAGTACAGCCCCAAAATGG, Rev: AACAAAGTCTGGCCTGTATCCAA). Each bar represents mean expression±s.e.m., compared with control of eight animals per genotype. (**c**) Location of the highly conserved miR-206-binding site within *Sfrp1* 3′UTR. TargetScanMouse was used to identify putative miRNA-binding sites in the 3′UTR region of *Sfrp1*. The level of conservation among mammals was assessed using information from TargetScan and the conservation track of the UCSC Genome Browser, which was also used to extract sequence information (http://genome.ucsc.edu/: mm9 assembly)^72^. ClustalW2 was used to align sequences to demonstrate nucleotide level conservation. (**d**) miR-206 can repress the *Sfrp1* 3′UTR and this is diminished upon mutation of the miR-206-binding site. To assess if miR-206 targeted the *Sfrp1* 3′UTR, HeLa cells were transfected with 200 ng of appropriate pSG5 luciferase vector and 50 ng of pSG5 Renilla using Lipofectamine 2000 (Thermo Fisher Scientific). To overexpress miR-206, an additional 200 ng molar equivalent of either pMMTV or pMMTV miR-206 was also used. For all assays, addition of pUC19 DNA was used to ensure all transfections had equal DNA content. Luciferase readings were assessed 48 h post transfection using a DTX 880 Multimode plate reader (Beckman Coulter, Brea, CA, USA) and normalized to Renilla readings. Each bar represents the mean±s.e.m. of three biological replicates. (**e**) *Sfrp1* expression is reduced in MMTV miR-206 glands at day 1 of lactation and in aged animals. The expression of *Sfrp1* within mammary glands from FVB or MMTV miR-206 mice at day 1 of lactation or in aged mice (12–15 month old) was assessed using qRT-PCR as described above. Data represent *n*=3 animals per genotype. Statistical significance was determined by Student's *t*-test and denoted as ****P<0.0001, **P<0.01 and *P<0.05.

To further explore the *Brca1*-miR-206 pathway, we identified potential gene targets of miR-206 (Figure 4a). Four genes were shortlisted on the basis of the results from four independent miRNA target prediction programs—*Sfrp1*, *Daam1*, *Slc39a10* and *Zbtb4* (Figure 4a). To validate these predictions, we examined the levels of these genes in the mammary glands of *MMTV-Cre Brca1*^*Co/Co*^ mice. Only *Sfrp1* had reduced expression in the lactating mammary gland of *MMTV-Cre Brca1*^*Co/Co*^ mice (Figure 4b). Sfrp1 is a secreted antagonist of the wingless-type MMTV integration site (Wnt)-signaling pathway,^59^ whose expression is frequently lost in a variety of cancers, including breast cancer via promoter methylation.^60^ Repression of *SFRP1* is associated with poor prognosis with increasing stage of malignancy.^52, 61, 62, 63, 64, 65^ Although many reports have focused on assessing the methylation status of the *SFRP1* promoter, several studies have shown that some tumors exhibit decreased *SFRP1* expression, independent of promoter methylation,^52, 66^ suggesting additional regulatory mechanisms may be involved. In addition, miR-206 has been shown to regulate *Sfrp1* expression during myogenesis in a porcine model.^67^

A miR-206-binding site was identified in the *Sfrp1* 3′UTR and shown to be evolutionary conserved (Figure 4c). To determine whether miR-206 could repress the *Sfrp1* 3′UTR in mice, a luciferase reporter assay was conducted with the *Notch* 3′UTR, a known target of miR-206, as a positive control. Overexpression of miR-206, decreased the activity of both the *Sfrp1* and *Notch3* 3′UTRs (Figure 4d), confirming that miR-206 can repress both *Sfrp1* and *Notch3*. To further demonstrate that the reduced luciferase activity is a direct consequence of miR-206 targeting the predicted *Sfrp1* 3′UTR-binding site, mutations were introduced into the miRNA-binding site. Increased expression of miR-206 did not affect the activity of the mutated *Sfrp1* 3′UTR (Figure 4d), suggesting that the decrease in luciferase signal can be attributed to the binding of miR-206 to *Sfrp1* at the predicted binding site. We also found that *Sfrp1* expression was reduced in mice at day 1 of lactation and 12–15 months of age (Figure 4e). These results are consistent with the mammary epithelial phenotype observed and suggest that this phenotype is potentially a result of deregulated Wnt signaling. Though, there are conflicting reports of Sfrp1 function in the mammary gland, a recent study has shown that *Sfrp1* deficiency induces increased adiposity upon forced weight gain in mice, consistent with our observations in aged MMTV miR-206 mice. Another team demonstrated that the knockout of *Sfrp1* in mice mammary glands promotes precocious mammary gland development with branching and alveolar development characteristic of the midpregnant mammary gland,^68, 69^ a feature that has not been observed in the MMTV miR-206 mice (Figure 3a). A probable reason is that this phenotype has been masked by the upregulation of the *Igf-1*-signaling cascade.^55, 70, 71^

## Conclusions

In conclusion, this work has demonstrated that miR-206 is overexpressed in *Brca1*-deficient cells and that overexpression of miR-206, in several ways, mirrors the phenotype in *MMTV-Cre Brca1*^*Co/Co*^ mice. This raises the possibility that miR-206 contributes to the effects of *Brca1* loss in the mammary gland and that this likely involves the Wnt pathway through *Sfrp1* and the *Igf* pathways. Although we focused on miR-206, our data concur with other reports in suggesting other miRNAs, such as miR-155,^29^ can regulate downstream functions of *Brca1*, highlighting that this and other miRNAs are an important facet of *Brca1* biology and function. Understanding these mechanisms will be valuable for identifying new biomarkers and therapeutic targets and strategies for BRCA1-associated breast cancer.
