# Supplementary figures and images for: MicroRNA-206 is differentially expressed in Brca1-deficient mice and regulates epithelial and stromal cell compartments of the mouse mammary gland
Source: Oncogenesis. 2016 Apr 4;5(4):e218–. doi: 10.1038/oncsis.2016.27 (PMC4848838; doi:10.1038/oncsis.2016.27)

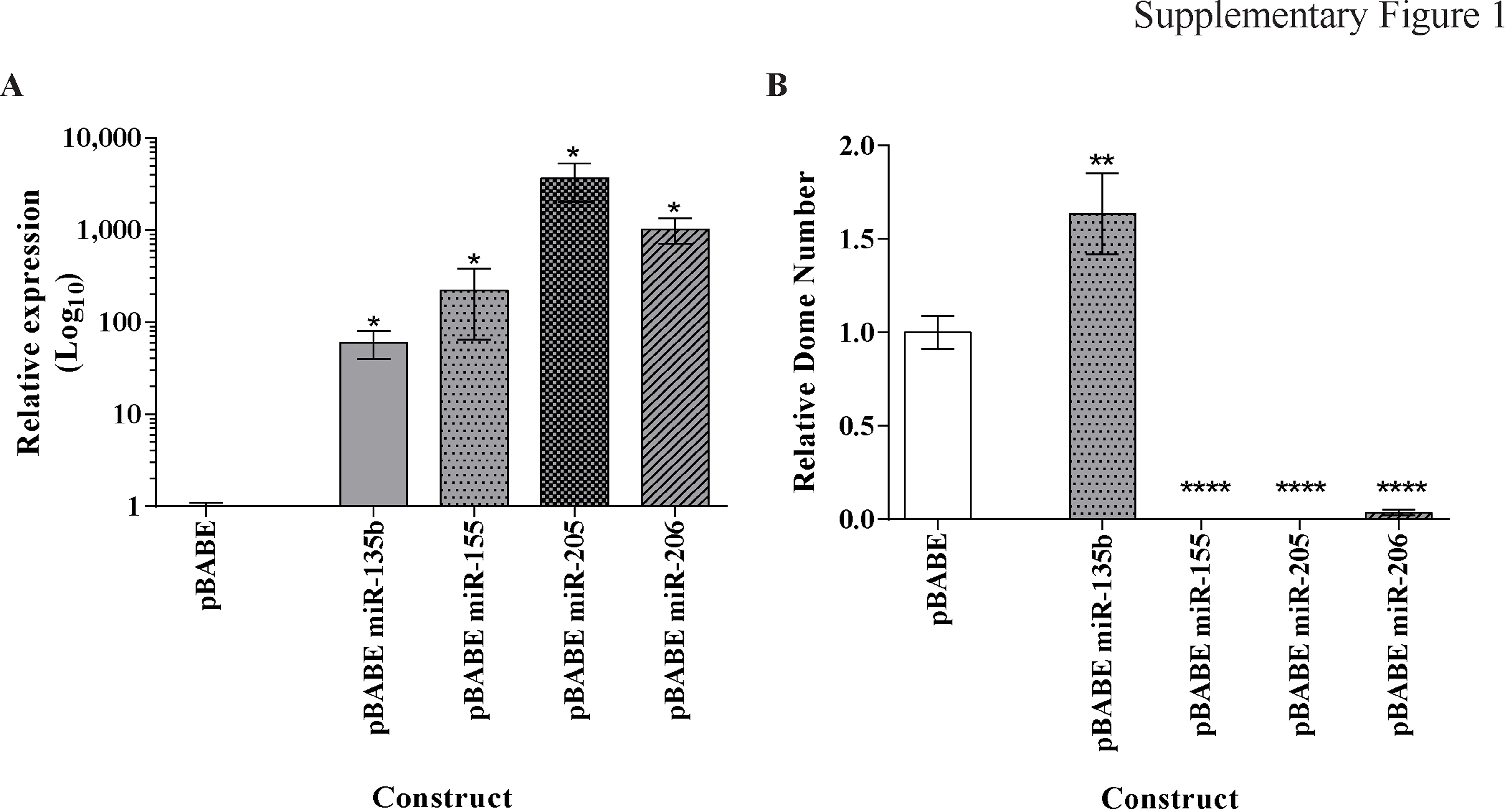

Supplement: Supplementary Figure 1 [file oncsis201627x1.tif]
