# Supplementary material for: MicroRNA-206 is differentially expressed in Brca1-deficient mice and regulates epithelial and stromal cell compartments of the mouse mammary gland
Source: Oncogenesis. 2016 Apr 4;5(4):e218–. doi: 10.1038/oncsis.2016.27 (PMC4848838; doi:10.1038/oncsis.2016.27)
Supplement: Supplementary Tables [file oncsis201627x2.pdf]

Supplementary Table 1 Predicted mRNA targets of differentially expressed miRNAs using m3RNA. Note: a higher WSP score corresponds to a stronger prediction.

miR-31				
**Symbol**	**Entrez Gene Name**	**Location**	**Type(s)**	**WSP score**
POU2F3	POU class 2 homeobox 3	Nucleus	transcription regulator	1.91E-01
IL34	interleukin 34	Extracellular Space	other	1.40E-01
PPP6C	protein phosphatase 6, catalytic subunit	Nucleus	phosphatase	1.28E-01
Cops2	COP9 (constitutive photomorphogenic) homolog, subunit 2 (Arabidopsis thaliana)	Nucleus	transcription regulator	1.20E-01
SLC25A28	solute carrier family 25 (mitochondrial iron transporter), member 28	Cytoplasm	other	1.10E-01
MOSPD2	motile sperm domain containing 2	Other	other	1.06E-01
MAP4K5	mitogen-activated protein kinase kinase kinase kinase 5	Cytoplasm	kinase	1.06E-01
TTR	transthyretin	Extracellular Space	transporter	1.05E-01
KANK1	KN motif and ankyrin repeat domains 1	Nucleus	transcription regulator	1.03E-01
RSBN1	round spermatid basic protein 1	Nucleus	other	1.02E-01
SH2D1A	SH2 domain containing 1A	Cytoplasm	other	1.00E-01
IFI30	interferon, gamma-inducible protein 30	Cytoplasm	enzyme	9.48E-02
TACC2	transforming, acidic coiled-coil containing protein 2	Nucleus	other	9.46E-02
STK40	serine/threonine kinase 40	Cytoplasm	kinase	9.19E-02
ATL2	atlastin GTPase 2	Cytoplasm	other	9.09E-02
DUSP7	dual specificity phosphatase 7	Cytoplasm	phosphatase	9.04E-02
UBN1	ubinuclein 1	Nucleus	transcription regulator	9.02E-02
NUP153	nucleoporin 153kDa	Nucleus	transporter	8.98E-02
TOPBP1	topoisomerase (DNA) II binding protein 1	Nucleus	other	8.97E-02
CYC1	cytochrome c-1	Cytoplasm	enzyme	8.95E-02

Supplementary Table 1

miR-135b				
**Symbol**	**Entrez Gene Name**	**Location**	**Type(s)**	**WSP score**
CPLX2	complexin 2	Cytoplasm	other	2.57E-01
TNPO1	transportin 1	Nucleus	transporter	2.25E-01
Esrra	estrogen related receptor, alpha	Nucleus	transcription regulator	2.24E-01
SP3	Sp3 transcription factor	Nucleus	transcription regulator	2.19E-01
ZNF652	zinc finger protein 652	Other	other	2.12E-01
NPAT	nuclear protein, ataxia-telangiectasia locus	Nucleus	transcription regulator	2.07E-01
DHODH	dihydroorotate dehydrogenase (quinone)	Cytoplasm	enzyme	2.04E-01
SYT2	synaptotagmin II	Cytoplasm	transporter	2.00E-01
DTNA	dystrobrevin, alpha	Plasma Membrane	other	1.97E-01
Gcg	glucagon	Cytoplasm	other	1.95E-01
MED13	mediator complex subunit 13	Nucleus	transcription regulator	1.92E-01
KCNAB3	potassium channel, voltage gated subfamily A regulatory beta subunit 3	Plasma Membrane	ion channel	1.90E-01
Foxn3	forkhead box N3	Other	other	1.90E-01
NR3C2	nuclear receptor subfamily 3, group C, member 2	Nucleus	ligand-dependent nuclear receptor	1.87E-01
VLDLR	very low density lipoprotein receptor	Plasma Membrane	transporter	1.71E-01
Skor1	SKI family transcriptional corepressor 1	Nucleus	transcription regulator	1.71E-01
PELI2	pellino E3 ubiquitin protein ligase family member 2	Cytoplasm	other	1.60E-01
FRMPD4	FERM and PDZ domain containing 4	Other	other	1.52E-01
NUP153	nucleoporin 153kDa	Nucleus	transporter	1.52E-01
ZDHHC6	zinc finger, DHHC-type containing 6	Nucleus	enzyme	1.50E-01

Supplementary Table 1

miR-148a				
**Symbol**	**Entrez Gene Name**	**Location**	**Type(s)**	**WSP score**
MEOX2	mesenchyme homeobox 2	Nucleus	transcription regulator	2.89E-01
CAND1	cullin-associated and neddylation-dissociated 1	Cytoplasm	transcription regulator	2.79E-01
S1PR1	sphingosine-1-phosphate receptor 1	Plasma Membrane	G-protein coupled receptor	2.70E-01
SOS2	son of sevenless homolog 2 (Drosophila)	Cytoplasm	other	2.55E-01
RC3H2	ring finger and CCCH-type domains 2	Plasma Membrane	enzyme	2.43E-01
XPO4	exportin 4	Nucleus	transporter	2.19E-01
TBX3	T-box 3	Nucleus	transcription regulator	2.10E-01
INO80	INO80 complex subunit	Nucleus	enzyme	2.06E-01
TNRC6A	trinucleotide repeat containing 6A	Nucleus	other	2.04E-01
ZBTB8A	zinc finger and BTB domain containing 8A	Nucleus	other	1.97E-01
ATP7A	ATPase, Cu++ transporting, alpha polypeptide	Plasma Membrane	transporter	1.93E-01
ROBO1	roundabout guidance receptor 1	Plasma Membrane	transmembrane receptor	1.90E-01
EML2	echinoderm microtubule associated protein like 2	Cytoplasm	other	1.85E-01
SCAF11	SR-related CTD-associated factor 11	Nucleus	other	1.82E-01
TNRC6C	trinucleotide repeat containing 6C	Cytoplasm	other	1.74E-01
KIAA0232	KIAA0232	Extracellular Space	other	1.72E-01
ELAVL4	ELAV like neuron-specific RNA binding protein 4	Cytoplasm	other	1.70E-01
ABCB7	ATP-binding cassette, sub-family B (MDR/TAP), member 7	Cytoplasm	transporter	1.58E-01
STRADB	STE20-related kinase adaptor beta	Cytoplasm	kinase	1.56E-01
USP32	ubiquitin specific peptidase 32	Cytoplasm	other	1.52E-01

Supplementary Table 1

miR-155				
**Symbol**	**Entrez Gene Name**	**Location**	**Type(s)**	**WSP score**
JARID2	jumonji, AT rich interactive domain 2	Nucleus	transcription regulator	3.14E-01
TSHZ3	teashirt zinc finger homeobox 3	Nucleus	transcription regulator	2.76E-01
FGF7	fibroblast growth factor 7	Extracellular Space	growth factor	2.47E-01
ADD3	adducin 3 (gamma)	Cytoplasm	other	2.06E-01
TRIP13	thyroid hormone receptor interactor 13	Cytoplasm	transcription regulator	1.84E-01
LIN9	lin-9 DREAM MuvB core complex component	Nucleus	other	1.69E-01
AICDA	activation-induced cytidine deaminase	Cytoplasm	enzyme	1.68E-01
WEE1	WEE1 G2 checkpoint kinase	Nucleus	kinase	1.65E-01
ZNF652	zinc finger protein 652	Other	other	1.40E-01
ARID2	AT rich interactive domain 2 (ARID, RFX-like)	Nucleus	transcription regulator	1.38E-01
BACH1	BTB and CNC homology 1, basic leucine zipper transcription factor 1	Nucleus	transcription regulator	1.37E-01
HIVEP2	human immunodeficiency virus type I enhancer binding protein 2	Nucleus	transcription regulator	1.34E-01
PICALM	phosphatidylinositol binding clathrin assembly protein	Cytoplasm	other	1.34E-01
NR2F2	nuclear receptor subfamily 2, group F, member 2	Nucleus	ligand-dependent nuclear receptor	1.31E-01
GHR	growth hormone receptor	Plasma Membrane	transmembrane receptor	1.29E-01
ACTA1	actin, alpha 1, skeletal muscle	Cytoplasm	other	1.27E-01
ZIC3	Zic family member 3	Nucleus	transcription regulator	1.27E-01
RC3H2	ring finger and CCCH-type domains 2	Plasma Membrane	enzyme	1.26E-01
PEA15	phosphoprotein enriched in astrocytes 15	Cytoplasm	transporter	1.23E-01
CACNB4	calcium channel, voltage-dependent, beta 4 subunit	Plasma Membrane	ion channel	1.23E-01

Supplementary Table 1

miR-181c				
**Symbol**	**Entrez Gene Name**	**Location**	**Type(s)**	**WSP score**
KLF6	Kruppel-like factor 6	Nucleus	transcription regulator	3.08E-01
EIF4A2	eukaryotic translation initiation factor 4A2	Cytoplasm	translation regulator	2.75E-01
TNPO1	transportin 1	Nucleus	transporter	2.75E-01
GNAQ	guanine nucleotide binding protein (G protein), q polypeptide	Plasma Membrane	enzyme	2.73E-01
BTBD3	BTB (POZ) domain containing 3	Other	other	2.28E-01
NR6A1	nuclear receptor subfamily 6, group A, member 1	Nucleus	ligand-dependent nuclear receptor	2.14E-01
CPEB4	cytoplasmic polyadenylation element binding protein 4	Plasma Membrane	other	2.13E-01
ZBTB4	zinc finger and BTB domain containing 4	Nucleus	transcription regulator	2.01E-01
GPBP1	GC-rich promoter binding protein 1	Nucleus	transcription regulator	1.99E-01
RLF	rearranged L-myc fusion	Nucleus	transcription regulator	1.97E-01
FIGN	fidgetin	Nucleus	other	1.93E-01
BRAF	B-Raf proto-oncogene, serine/threonine kinase	Cytoplasm	enzyme	1.90E-01
EPC2	enhancer of polycomb homolog 2 (Drosophila)	Other	other	1.88E-01
SS18L1	synovial sarcoma translocation gene on chromosome 18-like 1	Nucleus	transcription regulator	1.86E-01
SPRY4	sprouty RTK signaling antagonist 4	Plasma Membrane	other	1.80E-01
CCNF	cyclin F	Nucleus	other	1.76E-01
PCDHAC2	protocadherin alpha subfamily C, 2	Plasma Membrane	other	1.70E-01
ZIC2	Zic family member 2	Nucleus	transcription regulator	1.67E-01
INO80D	INO80 complex subunit D	Other	other	1.61E-01
PLCL2	phospholipase C-like 2	Cytoplasm	enzyme	1.61E-01

Supplementary Table 1

miR-200b				
**Symbol**	**Entrez Gene Name**	**Location**	**Type(s)**	**WSP score**
ZFPM2	zinc finger protein, FOG family member 2	Nucleus	transcription regulator	4.09E-01
ZEB1	zinc finger E-box binding homeobox 1	Nucleus	transcription regulator	3.40E-01
HNRNPD	heterogeneous nuclear ribonucleoprotein D (AU-rich element RNA binding protein 1, 37kDa)	Nucleus	transcription regulator	3.38E-01
ZEB2	zinc finger E-box binding homeobox 2	Nucleus	transcription regulator	2.99E-01
GPM6A	glycoprotein M6A	Plasma Membrane	ion channel	2.90E-01
ASAP1	ArfGAP with SH3 domain, ankyrin repeat and PH domain 1	Plasma Membrane	other	2.87E-01
SYNJ1	synaptojanin 1	Cytoplasm	phosphatase	2.51E-01
TBC1D12	TBC1 domain family, member 12	Extracellular Space	other	2.51E-01
HS3ST1	heparan sulfate (glucosamine) 3-O-sulfotransferase 1	Cytoplasm	enzyme	2.50E-01
NFIA	nuclear factor I/A	Nucleus	transcription regulator	2.48E-01
SLIT2	slit guidance ligand 2	Extracellular Space	other	2.46E-01
HNF1B	HNF1 homeobox B	Nucleus	transcription regulator	2.44E-01
RND3	Rho family GTPase 3	Cytoplasm	enzyme	2.44E-01
DUSP1	dual specificity phosphatase 1	Nucleus	phosphatase	2.43E-01
REV1	REV1, polymerase (DNA directed)	Nucleus	enzyme	2.42E-01
NOG	noggin	Extracellular Space	growth factor	2.38E-01
PI4KB	phosphatidylinositol 4-kinase, catalytic, beta	Cytoplasm	kinase	2.35E-01
SEC23A	Sec23 homolog A, COPII coat complex component	Cytoplasm	transporter	2.31E-01
NALCN	sodium leak channel, non selective	Plasma Membrane	ion channel	2.27E-01
COPS8	COP9 signalosome subunit 8	Nucleus	other	2.25E-01

Supplementary Table 1

miR-205				
**Symbol**	**Entrez Gene Name**	**Location**	**Type(s)**	**WSP score**
VASN	vasorin	Plasma Membrane	other	2.48E-01
MGRN1	mahogunin ring finger 1, E3 ubiquitin protein ligase	Cytoplasm	enzyme	2.02E-01
CHN1	chimerin 1	Cytoplasm	other	1.89E-01
RBM47	RNA binding motif protein 47	Nucleus	other	1.70E-01
NFAT5	nuclear factor of activated T-cells 5, tonicity-responsive	Nucleus	transcription regulator	1.39E-01
SRSF10	serine/arginine-rich splicing factor 10	Nucleus	other	1.35E-01
CADM1	cell adhesion molecule 1	Plasma Membrane	other	1.31E-01
PHC2	polyhomeotic homolog 2 (Drosophila)	Nucleus	other	1.30E-01
NAA25	N(alpha)-acetyltransferase 25, NatB auxiliary subunit	Cytoplasm	other	1.28E-01
INPPL1	inositol polyphosphate phosphatase-like 1	Cytoplasm	phosphatase	1.28E-01
PABPC1L	poly(A) binding protein, cytoplasmic 1-like	Cytoplasm	other	1.22E-01
HS3ST1	heparan sulfate (glucosamine) 3-O-sulfotransferase 1	Cytoplasm	enzyme	1.12E-01
Ank2	ankyrin 2, brain	Plasma Membrane	other	1.09E-01
SLC35B3	solute carrier family 35 (adenosine 3'-phospho 5'-phosphosulfate transporter), member B3	Cytoplasm	other	1.08E-01
LRP1	low density lipoprotein receptor-related protein 1	Plasma Membrane	transmembrane receptor	1.08E-01
CASC4	cancer susceptibility candidate 4	Cytoplasm	other	1.07E-01
ERRFI1	ERBB receptor feedback inhibitor 1	Cytoplasm	other	1.07E-01
SLC35A1	solute carrier family 35 (CMP-sialic acid transporter), member A1	Cytoplasm	transporter	1.06E-01
ESRRG	estrogen-related receptor gamma	Nucleus	ligand-dependent nuclear receptor	1.05E-01
PRPF8	pre-mRNA processing factor 8	Nucleus	other	1.04E-01

Supplementary Table 1

miR-206				
**Symbol**	**Entrez Gene Name**	**Location**	**Type(s)**	**WSP score**
CNN3	calponin 3, acidic	Cytoplasm	other	2.75E-01
LRCH1	leucine-rich repeats and calponin homology (CH) domain containing 1	Other	other	2.59E-01
CLCN3	chloride channel, voltage-sensitive 3	Plasma Membrane	ion channel	2.49E-01
DDX5	DEAD (Asp-Glu-Ala-Asp) box helicase 5	Nucleus	enzyme	2.36E-01
PPIB	peptidylprolyl isomerase B (cyclophilin B)	Cytoplasm	enzyme	2.27E-01
GJA1	gap junction protein, alpha 1, 43kDa	Plasma Membrane	transporter	2.20E-01
IP6K2	inositol hexakisphosphate kinase 2	Cytoplasm	kinase	2.13E-01
SEC23B	Sec23 homolog B, COPII coat complex component	Extracellular Space	transporter	1.96E-01
STC2	stanniocalcin 2	Extracellular Space	other	1.94E-01
Foxp1	forkhead box P1	Nucleus	transcription regulator	1.91E-01
CPEB1	cytoplasmic polyadenylation element binding protein 1	Cytoplasm	translation regulator	1.90E-01
TPPP	tubulin polymerization promoting protein	Cytoplasm	other	1.86E-01
SRSF9	serine/arginine-rich splicing factor 9	Nucleus	enzyme	1.83E-01
Msantd2	Myb/SANT-like DNA-binding domain containing 2	Other	other	1.82E-01
Ktn1	kinectin 1	Cytoplasm	other	1.78E-01
BDNF	brain-derived neurotrophic factor	Extracellular Space	growth factor	1.77E-01
CEBPZ	CCAAT/enhancer binding protein (C/EBP), zeta	Nucleus	transcription regulator	1.67E-01
EML3	echinoderm microtubule associated protein like 3	Other	other	1.64E-01
SERP1	stress-associated endoplasmic reticulum protein 1	Cytoplasm	other	1.62E-01
TRAPPC3	trafficking protein particle complex 3	Cytoplasm	other	1.58E-01

Supplementary Table 1

miR-210				
**Symbol**	**Entrez Gene Name**	**Location**	**Type(s)**	**WSP score**
ITGB5	integrin, beta 5	Plasma Membrane	other	1.92E-01
NEUROG3	neurogenin 3	Nucleus	transcription regulator	1.92E-01
Kcnn2	potassium intermediate/small conductance calcium-activated channel, subfamily N, member 2	Plasma Membrane	ion channel	1.16E-01
C6orf136	chromosome 6 open reading frame 136	Cytoplasm	other	1.09E-01
GNAO1	guanine nucleotide binding protein (G protein), alpha activating activity polypeptide O	Plasma Membrane	enzyme	1.08E-01
ZADH2	zinc binding alcohol dehydrogenase domain containing 2	Cytoplasm	enzyme	1.05E-01
ELFN2	extracellular leucine-rich repeat and fibronectin type III domain containing 2	Extracellular Space	other	9.81E-02
CCDC97	coiled-coil domain containing 97	Other	other	9.52E-02
ISCU	iron-sulfur cluster assembly enzyme	Cytoplasm	other	9.33E-02
B4GALT5	UDP-Gal:betaGlcNAc beta 1,4- galactosyltransferase, polypeptide 5	Cytoplasm	enzyme	9.09E-02
TMEM151B	transmembrane protein 151B	Other	other	9.07E-02
Tpm1	tropomyosin 1, alpha	Plasma Membrane	other	9.00E-02
Pdlim3	PDZ and LIM domain 3	Plasma Membrane	other	8.95E-02
NPTX1	neuronal pentraxin I	Extracellular Space	other	8.87E-02
HIF3A	hypoxia inducible factor 3, alpha subunit	Nucleus	transcription regulator	8.63E-02
GPD1L	glycerol-3-phosphate dehydrogenase 1-like	Cytoplasm	enzyme	8.57E-02
ENPP5	ectonucleotide pyrophosphatase/phosphodiesterase 5 (putative)	Extracellular Space	enzyme	8.41E-02
MID1IP1	MID1 interacting protein 1	Cytoplasm	other	8.38E-02
PPTC7	PTC7 protein phosphatase homolog	Cytoplasm	phosphatase	8.36E-02
FAM222A	family with sequence similarity 222, member A	Other	other	8.34E-02

Supplementary Table 2

In silico analysis of diseases, molecular and physiological functions potentially affected by gene expression differences in conditional *Brca1* knockout mice at day 1 of lactation.

**Processes influenced upon miRNA down-regulation & mRNA up-regulation**			**Processes influenced upon miRNA up-regulation & mRNA down-regulation**		
	p-value	# molecules		p-value	# molecules
**Diseases and Disorders**			**Diseases and Disorders**		
Cancer	9.20E-04	106	Cancer	1.20E-02	62
Organismal Injury and Abnormalities	9.20E-04	107	Organismal Injury and Abnormalities	1.32E-02	63
Cardiovascular Disease	8.97E-04	18	Renal and Urological Disease	8.78E-03	17
Tumor Morphology	8.10E-04	22	Endocrine System Disease	8.96E-03	19
Connective Tissue Disorders	9.27E-04	66	Gastrointestinal Disease	9.68E-03	47
**Molecular and Cellular Functions**			**Molecular and Cellular Functions**		
Cell Death and Survival	9.35E-04	59	Cell-to-cell Signaling and Interaction	9.02E-03	10
Cellular Growth and Proliferation	8.39E-04	66	Carbohydrate Metabolism	1.32E-02	18
Cellular Movement	9.13E-04	47	Energy Production	1.02E-02	5
Cellular Development	8.39E-04	60	Lipid Metabolism	1.32E-02	10
Cellular Function and Maintenance	8.10E-04	53	Small Molecular Biochemistry	1.32E-02	18
**Physiological System Development and Function**			**Physiological System Development and Function**		
Organismal Survival	3.06E-04	49	Nervous System Development and Function	1.32E-02	12
Cardiovascular System Development & Function	8.47E-04	38	Organ Morphology	1.32E-02	25
Organismal Development	9.13E-04	67	Organismal Development	1.32E-02	27
Immune Cell Trafficking	9.13E-04	25	Hematological System Development and Function	9.91E-03	10
Tissue Morphology	8.52E-04	57	Tissue Development	1.32E-02	21
