# Supplementary material for: MicroRNA-206 is differentially expressed in Brca1-deficient mice and regulates epithelial and stromal cell compartments of the mouse mammary gland
Source: Oncogenesis. 2016 Apr 4;5(4):e218–. doi: 10.1038/oncsis.2016.27 (PMC4848838; doi:10.1038/oncsis.2016.27)
Supplement: Supplementary Information [file oncsis201627x3.docx]

**Supplementary Figure 1. Over-expression of the Brca1 associated miRNAs miR-155, miR-205 and miR-206 reduces the ability of HC11 mouse mammary epithelial cells to form dome structures *in vitro.***

HC11 cells were transduced with pBABE or pBABE miR-135b, miR-155, miR-205 and miR-206 viruses, created by transfecting BOSC23 cells and stable, expressing cell lines selected for using Puromycin (Clontech Laboratories, Mountain View, CA, U.S.A). Cells were then plated into the HC11 Dome Assay. Sub-confluent cells in a 6 well format were cultured in recombinant EGF-free HC11 media for 48 hours. The media was then changed to include 100 nM dexamethasone and 5 μg/ml ovine prolactin (both from Sigma-Aldrich, St Louis, MO, U.S.A) in rEGF-free HC11 media for a further 72 days. During this time, the media was replaced every 48 hours. The number of domes was then counted manually, as observed using a light microscope on day 8 of the assay. Each bar represents mean expression ± SEM, compared to control of three independent experiments.

**Supplementary Table 1. Predicted mRNA targets of differentially expressed miRNAs using m3RNA**.

The predicted targets of nine differentially expressed mouse miRNAs were assessed using m3RNA (31) with default settings. The WSP (Weighted Score of Precision) was used to identify the top 20 genes that were strongest predictions on the basis of multiple algorithms and experimental evidence (31). A higher WSP score corresponds to a stronger prediction. Gene names were then entered into Ingenuity Pathway Analysis to pull out Entrez Gene Names, Subcellular localization and general function of the gene.

**Supplementary Table 2.**

***In Silico* analysis of diseases, molecular and physiological functions potentially affected by gene expression differences in conditional *Brca1* knockout mice at day 1 of lactation.**

The mRNA targets of miRNAs over-expressed in conditional *Brca1* knockout mice were evaluated using Ingenuity Pathway Analysis (IPA). These were then compared to the gene list of mRNAs downregulated at day one of lactation (11) and any genes that were both a miRNA target and downregulated were used for a Core Analysis using IPA. The top five diseases and disorders, molecular and cellular functions and physiological system development and function pathways are displayed with the p-value and number of molecular in each category. The inverse was also assessed, with miRNAs that were down-regulated and mRNAs that were up-regulated in conditional *Brca1* knockout mammary glands at day one of lactation.
